# Are completed ReSPECT plans facilitating person-centred care? An evaluation of completed plans in UK general practice

**DOI:** 10.1016/j.resplu.2024.100780

**Published:** 2024-09-21

**Authors:** Caroline J. Huxley, Karin Eli, Claire A. Hawkes, Frances Griffiths, Martin Underwood, Gavin D. Perkins, Hazel Blanchard, Jenny Harlock, Julia Walsh, Anne-Marie Slowther

**Affiliations:** a Warwick Medical School, University of Warwick, Gibbet Hill, Coventry CV4 7AL, United Kingdom; b Florence Nightingale Faculty of Nursing, Midwifery and Palliative Care, King’s College London, James Clerk Maxwell Building, 57 Waterloo Road, London SE1 8AW, United Kingdom; c Forrest Medical Centre, 6 Prior Deram Walk, Coventry CV4 8FT, United Kingdom

**Keywords:** Recommended Summary Plan for Emergency Care and Treatment (ReSPECT), Emergency care treatment plans (ECTPs), Person-centred care

## Abstract

**Background:**

The Recommended Summary Plan for Emergency Care and Treatment (ReSPECT) includes agreed clinical recommendations for a person’s care in a future emergency which have been informed by discussion of the person’s preferences. Previous evaluation of ReSPECT plans in acute NHS hospitals found inconsistencies in recording patient’s preferences and involvement in the plan, and infrequent justification for recommendations.

**Aim:**

To explore to what extent ReSPECT recommendations reflect individual preferences, as documented in the plan.

**Methods:**

ReSPECT plans of adults were collected from 11 General Practices in England. We adapted an evaluation tool used previously to analyse ReSPECT plans in acute settings. Free text sections for individual values/preferences and clinical recommendations were examined for clarity, consistency and congruency between them.

**Results:**

We retrieved 141 ReSPECT plans. Patients or those close to the patient were recorded as being consulted in most plans (94%). Individual preferences were completed in 57% of plans. Clinical recommendations reflected individual preferences by directly referencing the person and their preferences (31%), by being consistent with the documented preferences (30%), or by using the same wording as the preferences (6%).

**Conclusion:**

While many clinical recommendations reflect individual preferences, the preferences themselves are only recorded in just over half of ReSPECT plans. This is problematic, because the recording of individual preferences facilitates person-centred care, both directly by informing recommendations and indirectly when used to guide decision-making in situations not anticipated in the plan. Future training for clinicians should emphasize the need to document the personal values section of the plan.

## Introduction

**Fig. 1:**
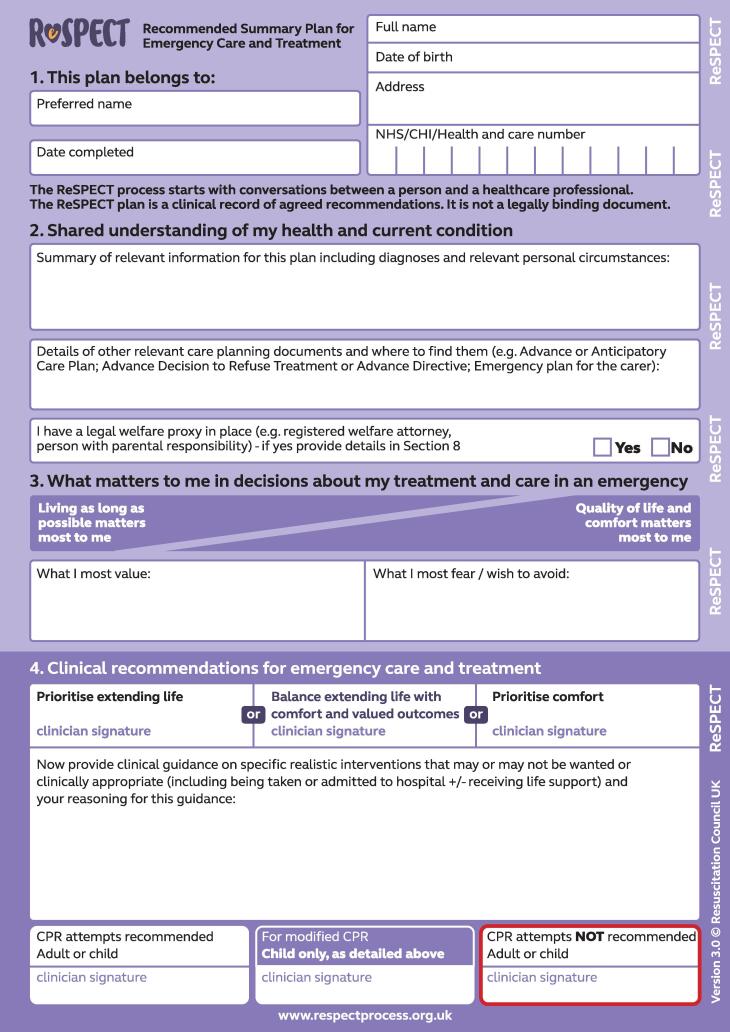

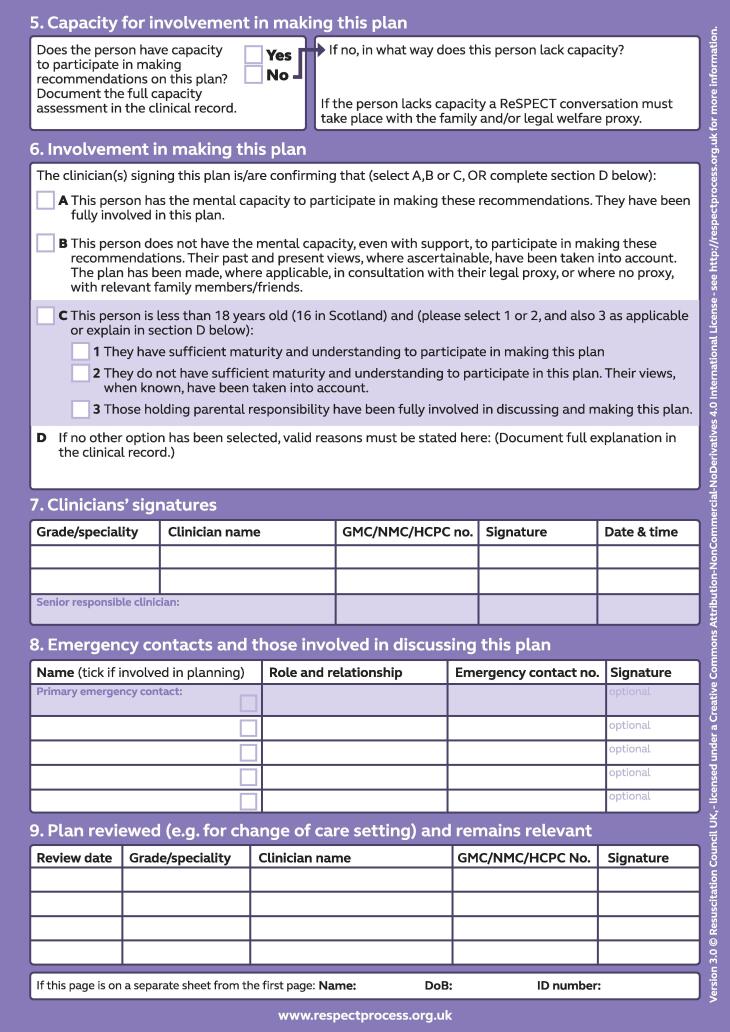
ReSPECT plan version 3.

**Fig. 2:**
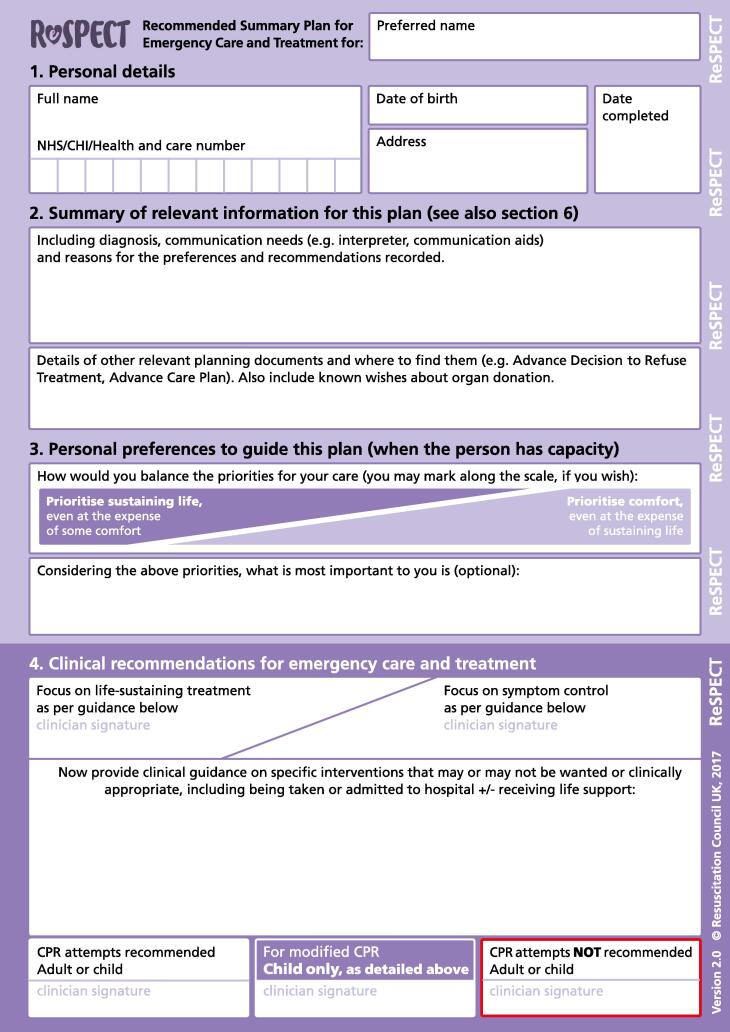

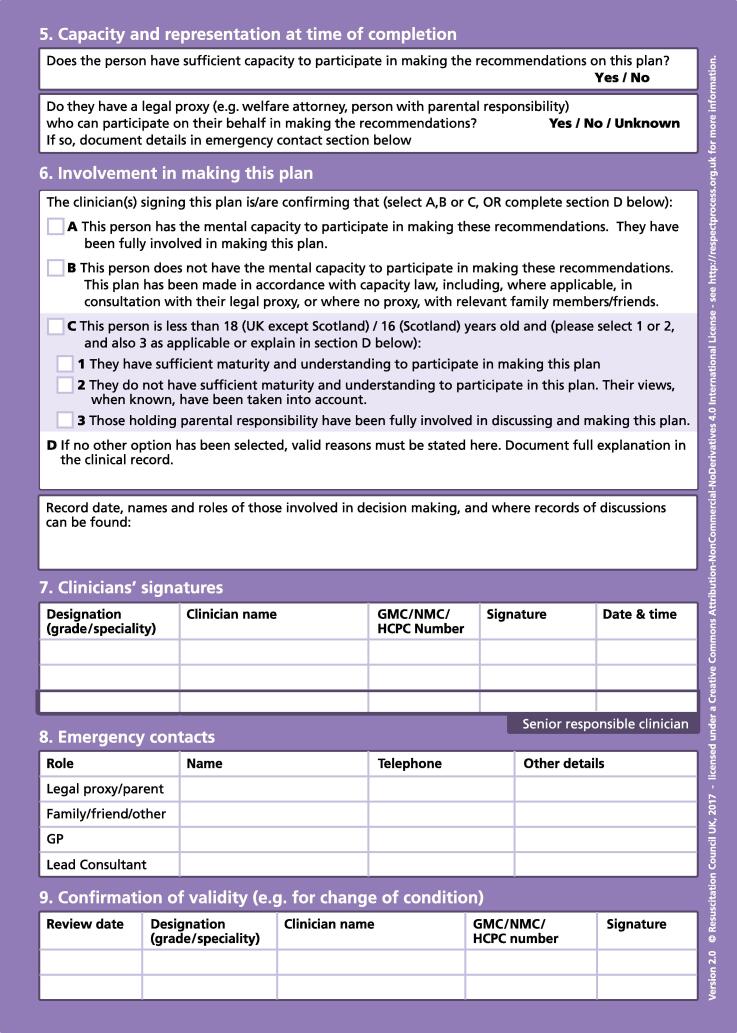
ReSPECT plan version 2.

Emergency care treatment plans (ECTPs) record treatment recommendations to be considered in an emergency when a person cannot make decisions for themselves. Their use has been described in both the UK and in North America with variation in aim and format across different models.^1^ The Recommended Summary Plan for Emergency Care and Treatment (ReSPECT) is the most widely used ECTP in the UK.^2^, ^3^ The aim of ReSPECT is to situate cardiopulmonary resuscitation (CPR) recommendations within a broader consideration of emergency treatment options, contextualising these with regard to the person’s health and values.^4^ The plan is patient-held and carried across healthcare settings with an expectation that it will be reviewed if there is a change in the patient’s circumstances. The ReSPECT process involves a conversation between a person (or someone close to them if they lack capacity) and their clinician about their values, preferences and fears regarding healthcare.^4^ This is documented on a ReSPECT plan. It includes space for clinicians to record the person’s relevant medical history, values and preferences for care, personalised clinical and CPR recommendations, the person’s capacity, and who was involved in creating the plan. Version 3 of the plan (see Fig. 1) was introduced in 2020, version 2 (see Fig. 2) was in use from 2017.^5^ Version 3 was developed following feedback from healthcare professionals, patients and their families. The aim of the changes was to make the plan even more patient-centred. Changes from version 2 include: changed wording to emphasise the importance of recording information about the person’s personal circumstances, their understanding and their perspective; an option to record a clinical recommendation to balance extending life with comfort and valued outcomes, rather than the binary options of extending life /prioritising comfort, reflecting a more nuanced approach to decision-making; a prompt that when decisions are made without involving the patient, the reasons for this are clearly recorded; and an option for the patient/their legal proxy/family member to sign the plan to demonstrate they have been actively involved in the discussion.^6^ The RCUK provides guidance for clinicians on completing the plan.^7^

Involving people in conversations and decisions about their treatment and taking account of their values and preferences is an important part of person-centred care. It helps improve quality of care, and is a crucial part of ethical clinical decision-making.^8^, ^9^ ReSPECT and other ECTPs offer a mechanism to improve the person-centred focus of emergency medical treatment decisions by personalising anticipatory treatment recommendations. For this to be realised in practice it is important to be confident that recommendations in ECTPs truly reflect the person’s preferences and values, and that these are accurately documented.

Our previous evaluation of recorded ReSPECT plans in acute NHS Trusts found that not all plans documented the involvement of the person or a family member; there was inconsistent recording of the person’s values and preferences; and reasons underpinning clinical recommendations were missing from many plans.^2^, ^10^, ^11^ This evaluation was conducted soon after the introduction of ReSPECT so findings may have reflected a learning-curve for clinicians. Interviews with hospital clinicians and focus groups with General Practitioners (GPs), conducted as part of this evaluation, suggested that ReSPECT conversations and plan completion may be better carried out in primary care where there is more time for conversations and GPs have more established relationships with patients and their families.^12^, ^13^ However, the study also found that GPs and hospital doctors conceptualised the plan differently and recommendations did not always translate well across settings. ReSPECT is now used widely in primary and community care in the UK so it is important to further evaluate its use in this setting.^14^ We carried out a large mixed-methods study of ReSPECT use in primary and community care.^15^ As part of this we evaluated ReSPECT plans held on GP patient records, including plans completed in hospital and in the community. We report an overall evaluation of plan completion in the study final report.^15^ In this paper we specifically focus on the person-centred elements of the plan: whether the person’s values and preferences are recorded, whether clinical recommendations reflect these preferences, and whether the involvement of the person or their family is documented.

## Method

For this study we focussed on the following sections of completed plans: the personal preferences sections of the plan (version 2 ‘Section 3: Personal preferences to guide this plan’ / version 3 ‘Section 3: What matters to me in decisions about my treatment and care in an emergency’); the clinical recommendations (versions 2 and 3 ‘Section 4: Clinical recommendations for emergency care and treatment’); and the patient’s capacity (version 2 ‘Section 5: Capacity and representation at time of completion’ / version 3 ‘Section 5: Capacity for involvement in making this plan’) and who was involved in creating the plan (versions 2 and 3 ‘Section 6: Involvement in making this plan’).

### Sampling and recruitment

Our sample included all completed ReSPECT plans for eligible patients retrievable from the general practitioner record in thirteen National Health Service (NHS) GP practices who participated in the wider research study.^15^ Practices were located in three regions in England, with diversity in levels of deprivation, urban/rural areas and ethnicity mix of the local population.

**Fig. 3:**
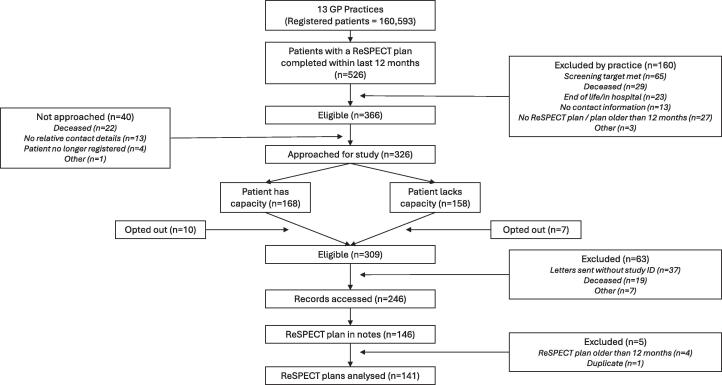
Flow chart of ReSPECT plan retrieval across study populations.

Each site identified patients who were over 18 years old and recorded as having a ReSPECT plan completed within the preceding 12 months. Patients known to be in the final stages of a terminal illness or currently in hospital were excluded to minimise distress. We sent eligible patients, or the next of kin of patients who lacked capacity, information about the study (including that we would access their medical records) and provided a mechanism for them to opt out of the study if they wished (see Fig. 3).

### Data collection

Data collection commenced at least four weeks after the participant information letters were sent to allow time for patients (or their relatives) to opt out. The records of all eligible patients, excluding those who had opted out, were accessed by a study researcher (JW, JH, or AS), or member of practice staff, who identified whether a ReSPECT plan was included. Identified ReSPECT plans were either scanned or electronically copied, and identifiable information redacted before the data were securely transferred to the study team using NHS encrypted email. Data collection took place from February 2021 to January 2023. Both version 2 and version 3 ReSPECT plans were included.

### Data analysis

For our overall analysis of plan completion, we adapted a tool we had previously developed to assess the quality of ReSPECT plans completed in secondary care (see Supplementary Table S1 for original, and Supplementary Table S2 for adapted tool).^10^ The tool was based on accountability for reasonableness, which is an ethical decision-making framework that emphasises transparency of decision-making, with decisions based on reasons that stakeholders can agree are relevant.^16^ Each item on the ReSPECT plan was assigned a score to indicate whether that item had been completed, and for the clinical recommendations how detailed a response had been provided (a score of ‘2′ for a detailed response, ‘1′ for general instructions, and ‘0′ for no response). For each ReSPECT plan, the numerical data determined through the evaluation tool were entered into an Excel document, and this was used to calculate response frequencies. The tool was adapted to ensure suitability for use with both version 2 and 3 ReSPECT plans. A detailed description of this process of refinement and quality assurance is provided in our wider project report.^15^ Using the modified tool all plans in the dataset were analysed by CJH (research psychologist) with KE (medical anthropologist), CAH (nurse/emergency care researcher), AS (GP/clinical ethicist) and FG (GP/medical sociologist) each analysing 25% so that all plans were evaluated by two team members. Evaluations were discussed and compared to achieve consensus across scores. While there were some differences in wording between versions 2 and 3, the aim of each section and nature of individual items in ReSPECT versions 2 and 3 were comparable.

**Table 1:** Frequency of personal preferences (grouped into categories with illustrative text).

Preference categories (n = 80)	Version 2 (n = 70)	Version 3 (n = 10)
Comfort (n = 48, 59%)	n = 42 *“Comfort only”*	n = 6 Avoid *“Repeated hospital admissions* Values *“Prioritise comfort − passing without fuss or treatment”*
Remaining at home/with family (n = 40, 49%),	n = 34 *“To stay at home and values comfort over life sustaining treatments. Preferred place of death: home”*	n = 6 Avoid *“Hospital admission, uncertainty”* Values *“Being kept comfortable, familiar surroundings, husband and daughter present”*
Maintaining dignity (n = 9, 11%)	n = 8 *“To have privacy and dignity maintained. To be kept pain and distress free.”*	n = 1 Avoid “*Does not want anyone pressing on his chest to resuscitate him”* Values *“Preservation of my dignity and comfort”*
Quality of life (n = 11, 14%)	n = 10 *“Would rather prioritise quality of life and comfort. Preferred place of death: home”*	n = 1 Avoid “[left blank]” Values *“Quality of life and comfort matters most to me”*
Preferred place of death, typically their home, care home, or hospice (n = 41, 29%)	n = 41 “*Comfort and quality of life. Preferred place of death: care home. To remain in care home*.”	n = 0
Active treatments (n = 3, 4%)	n = 3 *“Would prefer to be admitted to hospital if required for further investigations and treatment. Preferred place of death: Undecided at present”*	n = 0

We analysed the free text responses to section 3 (personal preferences) and section 4 (clinical recommendations) as follows. For the free text in the ‘personal preferences’ section we used content analysis to identify the frequency of different preferences. Rather than focussing on frequency of use of specific words, we focussed on categories within preferences. Data from one plan could be coded to more than one category (e.g. the phrase “*Would prefer to be kept comfortable at home and avoid unnecessary hospital admission”* was categorised as preferences for both comfort and for remaining at home/with family). CJH and AS read all completed patient preferences sections with categories agreed and refined following discussion. CJH then categorised all text in completed sections into the agreed categories and AS confirmed categorisation with no discrepancies noted. For the clinical recommendations CJH read both scale and free text responses to section 3 (patient preferences) and text in section 4 (clinical recommendations) on all plans, and AS read 30%, to identify potential congruency. Following discussion three categories of congruency were identified between clinical recommendations and patient preferences/values. CJH then categorised the text from each plan using these categories and AS confirmed categorisation. Disagreement in two cases was discussed and resolved.

### Ethics considerations

The overall study was approved by the London South East Research Ethics Committee (21/LO/0455). Data were collected without individual consent with Confidentiality Advisory Group approval (CAG reference 21/CAG/0089). Participants were given an opportunity to opt out of the study before data collection commenced. Personal identifiers on the ReSPECT plans were redacted prior to transfer to the study team and a study identification number added. The pseudonymous records were transferred to the study team using an encrypted NHS platform. Records were stored in password-protected files on a secure server.

## Results

We identified 526 patients with a record of ReSPECT plan completion in the previous 12 months across the GP practices (0.3% of the total practice population across practices). Of these, 326 people met our inclusion criteria, 17 of whom opted out of the study, and 63 of whom were excluded. Of the remaining 246 we were able to retrieve 146 (59%) ReSPECT plans from 11 practices (see Fig. 3). Four retrieved plans were excluded because they were completed over 12 months prior to data collection, and one plan was duplicated, leaving 141 plans for analysis. Most plans (n = 102) were version 2 while the remainder (n = 39) were version 3. The age range of patients was 51 to 101 years (median = 86 years), and more were female (n = 96, 68%) than male (n = 45, 32%). Across the 11 practices from which we retrieved plans, the overall number of ReSPECT plans completed per 1,000 patients ranged from 0.93 to 8.0.

### Completion of personal preference sections

The preference scale was completed in 45% (n = 63) plans, and the free text in 57% (n = 80). Both sections were completed in 30% (n = 42), and both sections were blank in 28% (n = 40) plans. The patient preferences section was less consistently completed in version 3 plans, compared to version 2 (31% v 50% for the scale, and 26% v 69% for the free text). Analysis of the free text responses shows that in over half of the plans the patient expressed a preference for comfort (see Table 1).

Fourteen plans (18%) provided what appeared to be clinical recommendations rather than personal preferences in this section. For example, the preferences section for one person stated:

“*If end of life is imminent, palliative care and support to be provided in the care home. For illness which is potentially reversible, i.e. infection requiring IV therapy a hospital admission is still for consideration and a conversation with son and daughter would be required. Hospital admission for potential fractures also indicated.*”

### Congruity between the clinical recommendations and the recorded patient preferences

Most plans recorded free text recommendations for treatments other than CPR (n = 122, 87%). Of these, a majority (93/122, 76%) provided specific recommendations on what treatment should be provided in a range of situations (e.g. “*Oral antibiotics in community if needed. Hospital admission if unable to manage pain or after significant trauma if needed”*), while 23% (28/122) gave general instructions (e.g., “*Not for hospital admission*”). Of the 122 plans with clinical recommendations 58% (n = 70) included personal preference text, and 43% (n = 53) included a completed personal preference scale (of which 18 plans did not include any corresponding text). Our analysis identified three ways in which the clinical recommendations reflected patient preferences.

First, some clinical recommendations specifically referenced the patient’s preferences (38/122, 31%). For example, the clinical recommendations for one person stated:

*“[Patient] does not wish to be admitted to hospital for anything other than a suspected fracture which would require radiological assessment or for bleeding which cannot be controlled. Care within own home is preference…”*

Clinical recommendations written as the patient’s preferences usually focussed on treatment objectives (e.g. comfort) and hospital admission (e.g. “*Would prefer to be kept comfortable at home and avoid unnecessary hospital admission”*) rather than addressing a range of specific scenarios.

On some plans where the clinical recommendations referenced the patient’s preferences, the patient’s values section was blank (8/38, 21%). For example, on one plan where the personal preferences section was not completed, the clinical recommendations stated “*Not for CPR, ITU or ventilation. Emphasis on comfort & symptom control. Preferred place of care/death is [care home]”*.

Second, some clinical recommendations were consistent with the patient’s preferences, without making specific reference to them (36/122, 30%). This was noticeable in version 3 plans where the format requires clinicians to specify what the person both ‘values’ and ‘fears/wishes to avoid’. In the example below, the patient valued comfort and feared repeated hospital admissions, and these are reflected in the recommendations:

“*Admission to hospital only where there is an acute need requiring short term care* e.g. *IV antibiotics and IV fluids, unless [patient] refuses admission. Admission if trauma occurs* e.g. *fracture. If admitted, not for ITU, renal dialysis, prolonged invasive intubation. Ward based care only and returned to place of residence ASAP*”

Third, we found seven plans (6%) (all version 2) in which the personal preferences and clinical recommendations were completed using identical wording. For example, on one plan the following text was found in both the personal preferences and recommendations sections: “*Would prefer to be kept comfortable in the care home and avoid unnecessary hospital admissions where possible.*” Here the clinical recommendation is presented as the patient’s preference.

### Patient/family involvement in the plan

Most plans had a ticked box to confirm that the patient or someone close to them was involved in making the plan (n = 133, 94%). The patient’s capacity was recorded in 131 plans (93%), with 59 (45%) recorded as lacking capacity. Where patients lacked capacity, whether they had a legal proxy was recorded on 76% (45/59) plans, the involvement of someone close to them (relative or friend) was recorded on 98% (58/59) plans, and this person’s name and role was recorded on 42% (25/59) plans.

## Discussion

Our evaluation of completed ReSPECT plans showed that most plans documented clinical recommendations, but the personal preferences and values sections of the plan were much less likely to be completed and were sometimes framed as clinical recommendations. Most plans documented that the patient or someone close to them was involved in making the plan. When the patient lacked capacity the involvement of someone close to them was documented in almost all cases. However, the name of the person consulted was recorded in fewer than half of these plans. Where patient preferences were recorded, clinical recommendations reflected recorded preferences either directly or indirectly. In some plans clinical recommendations referred to patient preferences, even when the patient preferences section was not completed. This suggests that some discussion between the clinician and person (or their family) had taken place to establish their values and preferences.

Documentation of involvement of the patient, or their family when the patient lacked capacity, was much higher than in our previous study.^2^, ^10^ This is an encouraging finding. However, it is of concern that the name and role of the person consulted was recorded in fewer than half of the plans. Knowing who has been consulted is important because of the legal requirement in the UK to consult with someone close to the patient when making a decision/recommendation in the best interests of someone who lacks capacity.^17^, ^18^

Framing the recommendations as personal preferences reflects wider understandings among clinicians that one purpose of ReSPECT is to record what is important to the patient in relation to their treatment and care.^15^, ^19^ However, ReSPECT is not simply a record of a person’s wishes, but rather a summary record of discussion between a clinician, a person and/or someone close to them, which leads to agreed clinical recommendations. The summary nature of the plan, and the limited space available for documentation, creates an inherent challenge for those completing it; how to provide sufficient detail to inform decision-making about a hypothetical future scenario such that the attending clinician can be confident that the recommendations accurately reflect the patient’s preferences and values. Ideally, detailed documentation of the ReSPECT conversation informing the plan should be recorded in the clinical notes, where the rationale for recommendations could be articulated in more depth. However, unlike in hospital when the ReSPECT plan should be read together with the hospital clinical record, in a community setting the acute responder attending a patient in an emergency may not have access to the GP clinical record. In our qualitative study of the experiences of GPs and care home staff in using ReSPECT we found examples of uncertainty and conflict in interpreting ReSPECT recommendations in specific emergency situations.^15^, ^20^ The need to interpret recommendations in context will require knowledge of the patient’s preferences and values. Thus clearly and comprehensively completed plans should both inform an emergency clinician of specific agreed clinical recommendations and provide an understanding of the person’s preferences and values, to guide decision-making in situations not anticipated in the clinical recommendations. However the minimum necessary information for an ECTP to be useful in an emergency remains uncertain and further evaluation is needed to understand how patient values can and do influence outcomes.

Concerns about the extent to which ECTP recommendations reflect or are consistent with patient preferences have been raised in relation to the use of Physician Orders for Life Sustaining Treatment (POLST) in care home settings in the United States. POLST is used to record preferences about specific treatments, including CPR, hospitalization, admission to intensive care, and ventilation.^21^ POLST was intended for use with people with a life-limiting condition, who were at risk of a life threatening clinical event, but its use has become more widespread to document the CPR preferences of healthier populations.^21^ Concerns have been raised about how valid the preferences recorded on POLST are within the wide range of medical contexts that people without life limiting conditions may encounter.^21^, ^22^, ^23^ For example, many people may prefer to be briefly intubated if a course of mechanical ventilation would enable a return to their previous state of health, but not if they were unlikely to be extubated. POLST is not designed for such context-specific decision-making.^22^ ReSPECT differs from both POLST and DNACPR orders in the UK, by specifically requiring documentation of the person’s values, therefore there is an opportunity in ReSPECT development to mitigate these concerns by emphasising the link between personal preferences/values and future decision-making in different clinical contexts.

The non-specificity of the clinical recommendations in many of the plans we examined may reflect the difficulty clinicians experience in predicting future clinical scenarios in a long-term community care context.^12^ This uncertainty could also present a challenge for patients to express specific preferences around treatment options. To address these identified challenges of recording preferences for specific but as yet hypothetical clinical scenarios, we consider that a focus on values (what is important to someone in terms of their healthcare) rather than specific preferences (the healthcare options they most favour) as included in the most recent version of ReSPECT, may be more useful in a community setting.^24^ The revised wording in version 3 of the ReSPECT plan was introduced in 2020, with the aim of emphasising the person-centred objectives of the ReSPECT conversation and its record. The timing of our study meant that three quarters of ReSPECT plans in our sample were version 2, thus it is not possible to say whether the changes made will have the desired impact. However, it is worth noting that compared to version 2 plans, a smaller percentage of version 3 plans had a completed patient preferences section. Further research to evaluate completion and use of ReSPECT version 3 is needed.

## Strengths/limitations

This is the first evaluation of completed ReSPECT plans held in primary and community care. A strength is the inclusion of thirteen GP practices, which represent different geographical localities and serve diverse populations. This study is limited because we were only able to evaluate 30% of ReSPECT plans identified across the practices. Approximately half of patients either met our exclusion criteria or had died prior to data collection. Furthermore, we were unable to access many plans because of variation in how ReSPECT was implemented. Practices using paper-based, patient-held plans did not always retain a copy in the clinical record. Some GP practices recorded ReSPECT recommendations in the patient notes, but these did not form part of the analysis.^15^ Furthermore we did not check for congruency between completed plans and the patient notes. Future evaluations of completed ReSPECT plans should review clinical notes to compare the completed plan with the detailed notes about the underlying conversation. The small number of version 3 plans in our sample meant it was not possible to draw robust conclusions on whether the phraseology on this latest version improved the person-centred approach to ReSPECT planning. Finally, interpretation of the preference scale (and the briefer free-text responses) was not straightforward, as the lack of detail provided on the plan left a degree of uncertainty about the person’s preferences/values.

## Conclusions

Most ReSPECT plans include clinical recommendations other than CPR and record the involvement of the patient or someone close to them when completing the plan. However, it is unclear from the documentation whether patient preferences are consistently used to inform ReSPECT recommendations. This is problematic, because the recording of individual preferences facilitates person-centred care, both directly by informing recommendations and indirectly when they can be used to guide decision-making in situations not anticipated in the plan. We suggest that training for healthcare professionals using ReSPECT should emphasise the importance of completing the personal values section of the plan and consider how to effectively and explicitly link the values and preferences recorded to clinical recommendations when completing the plan.

## Funding

This study was funded by the National Institute for Health and Care Research (NIHR), Health and Social Care Delivery Research Programme (13/13/16). The views expressed are those of the authors and not necessarily those of the NIHR or the Department of Health and Social Care. The funders had no role in study design, data collection and analysis, decision to publish, or preparation of the manuscript.

## CRediT authorship contribution statement

**Caroline J. Huxley:** Writing – original draft, Formal analysis. **Karin Eli:** Writing – review & editing, Formal analysis. **Claire A. Hawkes:** Writing – review & editing, Funding acquisition, Formal analysis, Conceptualization. **Frances Griffiths:** Writing – review & editing, Supervision, Methodology, Funding acquisition, Formal analysis, Conceptualization. **Martin Underwood:** Writing – review & editing, Funding acquisition, Conceptualization. **Gavin D. Perkins:** Writing – review & editing, Funding acquisition, Conceptualization. **Hazel Blanchard:** Writing – review & editing, Funding acquisition, Conceptualization. **Jenny Harlock:** Writing – review & editing, Data curation. **Julia Walsh:** Writing – review & editing, Data curation. **Anne-Marie Slowther:** Writing – review & editing, Supervision, Methodology, Investigation, Funding acquisition, Formal analysis, Data curation, Conceptualization.

## Declaration of competing interest

The authors declare the following financial interests/personal relationships which may be considered as potential competing interests: GDP is a Vice President and a member of the ReSPECT subcommittee of the Resuscitation Council UK, a volunteer Director for the European Resuscitation Council and co-chair for the International Liaison Committee on Resuscitation, Editor for Resuscitation and Resuscitation Plus. GDP is supported by the National Institute for Health Research (NIHR) Applied Research Collaboration (ARC) West Midlands.

AS, FG, JH, CAH, GDP and MU have participated in other research projects funded by NIHR.

CAH was involved in the development of the ReSPECT process and a member of the Resuscitation Council UK ReSPECT Research and Evaluation working group.

MU is a co-investigator on grants funded by the Australian 10.13039/501100000925NHMRC and Norwegian MRC. He is a director and shareholder of Clinvivo Ltd that provides electronic data collection for health services research. He is a co-investigator on two current and one completed NIHR funded studies that have, or have had, additional support from 10.13039/100008894Stryker Ltd.

AS joined the ReSPECT Stakeholder Group convened by Resuscitation Council UK in March 2024.
