# Supplementary material for: Are completed ReSPECT plans facilitating person-centred care? An evaluation of completed plans in UK general practice
Source: Resusc Plus. 2024 Sep 21;20:100780. doi: 10.1016/j.resplu.2024.100780 (PMC11447405; doi:10.1016/j.resplu.2024.100780)
Supplement: Supplementary Data 2 [file mmc2.docx]

**Supplementary Table 2: Adapted ReSPECT form evaluation tool (and guidance in italics)**

| **ReSPECT Plan Version 2** | **ReSPECT Plan Version 3** |
| --- | --- |
| Study ID number | Study ID number |
| Handwritten/typed (*state*) | Handwritten/typed (*state*) |
| **Box 1**  Is the date completed *(score 1 if date is present, score 0 for no date)* | **Box 1**  Is the date completed *(score 1 if date is present, score 0 for no date)* |
| **Box 2**  Are previous and present conditions recorded? *(score 1 for conditions listed, score 0 for no conditions)*  Are reasons for the recommendations recorded? *(score 0 for no reasons; score 1 for reasons related to CPR; score 2 for additional reasons*  Are communications needs recorded? *(note: yes or no)*  Are details of other planning documents recorded? *(note: yes, no other planning docs, or blank)*  Are known wishes about organ donation recorded? *(note: yes or no)*  Any comments on Box 2 (e.g. are conditions vague?) | **Box 2**  Are medical conditions recorded? *(score 1 for conditions listed, 0 for no conditions)*  Are personal circumstances recorded? *(note: yes or no)*  Are details of other planning documents recorded? *(note: yes, no other planning docs, or blank)*  Question about legal proxy completed? *(score 1 if completed, score 0 if blank)*  Does this person have a legal proxy? *(note: yes or no)*  Any comments on Box 2 (e.g. are conditions vague?) |
| **Box 3**  Is the optional scale completed? *(score 1 if completed, score 0 if not completed)*  Is the optional free text box completed? *(score 1 if text in any box, score 0 for no text)*  What is most important to the patient? *(copy text on form, write 'blank' if nothing is written)* | **Box 3**  Is the scale completed? *(score 1 if completed, score 0 if blank)*  Is the free text box completed? *(score 1 if text in any box, 0 for no text)*  What does the patient value? *(copy text on form, write 'blank' if nothing is written)*  What does the patient fear/wish to avoid? *(copy text on form, write 'blank' if nothing is written)* |
| **Box 4**  Is there a clinician signature on the scale, either on focus on life-sustaining treatment or focus on symptom control? *(score 1 for a signature; score 0 for no signature)*  What priority box is the signature in? *(select from options)*  Are details about specific interventions provided? *(score 2 if instructions are detailed (e.g. reversible acute conditions) or refer to patient's wishes or if patient is for everything; score 1 for general instructions like 'not for hospital admission'; score 0 if no interventions discussed)*  What are the recommendations? *(copy text from form, write 'blank' if nothing is written)*  If reasons are provided in Box 2 (see above), is the recommendation consistent with the reasons provided in Box 2? *(score 1 if reasons consistent, score 0 for inconsistent or missing reasoning)*  Is there a signature in the CPR box? *(score 1 for a signature; score 0 for no signature, leave blank if CPR boxes missing from form)*  What CPR box is the signature in?  If the CPR item is not scored, please explain why  Any comments on Box 4 | **Box 4**  Is there a clinician signature in one of the three priority boxes? *(score 1 if signature present, score 0 if there is no signature)*  What priority box is the signature in? *(select from options)*  Are details about specific interventions provided? *(score 2 if instructions are detailed (e.g. reversible acute conditions) or refer to patient's wishes or if patient is for everything; score 1 for general instructions like 'not for hospital admission'; score 0 if no interventions discussed)*  What are the recommendations? *(copy text from form, write 'blank' if nothing is written)*  Is reasoning provided? *(score 1 if reasoning is given, score 0 for no reasoning)*  Is there a signature in the CPR box? *(score 1 for signature, score 0 for no signature, leave blank if CPR boxes missing from form)*  What CPR box is the signature in? *(select from options)*  If the CPR item is not scored, please explain why  Any comments on Box 4 |
| **Box 5**  Is the question about capacity completed? *(score 1 for capacity recorded; score 0 if capacity no recorded)*  >If the question about capacity is completed, does the patient have capacity? *(note: yes or no)*  Is the question about legal proxy completed? *(score 1 if completed; score 0 if not completed)* | **Box 5**  Is the question about capacity completed? *(score 1 if completed, score 0 if capacity not recorded)*  >If the question about capacity is completed, does the patient have capacity? *(note: yes or no)*  Is there an explanation on the form of how the patient lacks capacity? *(note: yes or no)*  How does the patient lack capacity? *(copy text on form)* |
| **Box 6**  Is item A, B, or D selected? PILOT: Is item A, C, or D selected? *(score 1 if selected, score 0 if blank)*  > If item D is selected, are valid reasons included? *(score 0 if yes, score -1 if not)*  Are the date, names and roles recorded? *(score 2 if date, name and role recorded; score 1 if only name or role recorded; score 0 if blank)*  >Is a relative or someone close to the patient specified in the roles? *(note: yes or no)*  >If unclear, please explain why  Is there an indication of where records of discussions can be found? *(score 1 if present, score 0 for no indication of records)* | **Box 6**  Is item A, B, or D selected? PILOT: Is item A, C, or D selected? *(score 1 if selected, score 0 if blank)*  > If item D is selected, are valid reasons included? *(score 0 if yes, -1 if not)* |
| **Box 7**  Who is listed in the top 2 lines of box 7? *(See guidance if you cannot state a role.)*  Are the signatures, including date (and time), completed? *(score 1 if completed, score 0 if any information is missing)*  If scored 0 what was missing?  Is grade/speciality and registration number included? *(score 1 if completed, score 0 if any information is missing)*  Is there an appropriate signature, including date (and time), in the senior responsible clinician line? *(score 1 if completed, score 0 if any information is missing)*  If scored 0 what was missing?  Is grade/speciality and registration number included? *(score 1 if completed, score 0 if any information is missing)*  Who is listed as the senior clinician? (*role*)  Does this form appear to have been completed in hospital? *(note: yes, no or unclear)* | **Box 7**  Who is listed in the top 2 lines of box 7? *(See guidance if you cannot state a role)*  Are the signatures, including date (and time), completed? *(score 1 if completed, score 0 if any information is missing)*  If scored 0 what was missing?  Is grade/speciality and registration number included? *(score 1 if completed, score 0 if any information is missing)*  Is there an appropriate signature, including date (and time), in the senior responsible clinician line? *(score 1 if completed, score 0 if any information is missing)*  If scored 0 what was missing?  Is grade/speciality and registration number included? *(score 1 if completed, score 0 if any information is missing)*  Who is listed as the senior clinician? (*role*)  Does this form appear to have been completed in hospital? *(note: yes, no or unclear)* |
| **Box 8**  Are emergency contacts recorded? *(note: yes or no)*  >If the patient has a legal proxy, has the proxy's identity been documented? *(score 0 if yes, score -1 if not)* | **Box 8**  Are emergency contacts recorded? *(note: yes or no)*  Are the names, roles and relationships recorded? *(score 1 if name, role or relationship recorded)*  >Is a relative or someone close to the patient specified in the roles? *(note: yes or no)*  >If the patient has a legal proxy, has the proxy's identity been documented? *(score 0 if yes, -1 if not)*  Has the optional primary emergency contact signature item been completed? *(note: yes or no)* |
| **Box 9**  Has the confirmation of validity been used? *(note: yes or no)* | **Box 9**  Has the form been reviewed? *(note: yes or no)* |

Reproduced with permission from Slowther et al. Using the recommended summary plan for emergency care and treatment in primary care; a mixed methods study. Southampton (UK): National Institute for Health and Care Research 2024 In press. This is an Open Access article distributed in accordance with the terms of the Creative Commons Attribution (CC BY 4.0) licence, which permits others to distribute, remix, adapt and build upon this work, for commercial use, provided the original work is properly cited. See: <https://creativecommons.org/licenses/by/4.0/>
